# Exploring the role of chitosan and curcumin-loaded chitosan nanoparticles against chronic toxoplasma infection in experimental mice

**DOI:** 10.1038/s41598-025-25252-5

**Published:** 2025-11-24

**Authors:** Abeer A. Khedr, Nashwa Hamad, Salwa Mahmoud Abd-Elrahman, Sara Salah Abdel-Hakeem, Ahmed Kamal Dyab, Mervat M. Khalifa, Wafaa G. Mahmoud

**Affiliations:** 1 Department of Parasitology, Faculty of Veterinary Medicine, New Valley University, New Valley, El-Khargah, 72511 Egypt; 2 Department of Pathology, Faculty of Veterinary Medicine, Assiut University, Assiut, 71515 Egypt; 3 Department of Parasitology, Faculty of Veterinary Medicine, Assiut University, Assiut, 71526 Egypt; 4 Parasitology Laboratory, Zoology and Entomology Department, Faculty of Science, Assiut University, Assiut, 71526 Egypt; 5 Department of Medical Parasitology, Faculty of Medicine, Assiut University, Assiut, 71515 Egypt; 6 Department of Parasitology, School of Veterinary Medicine, Badr University in Assiut, NewNasser City, Assiut, Egypt; 7 Department of Medical Parasitology Faculty of Medicine, Assiut University, Asyut, 71515 Egypt

**Keywords:** *Toxoplasma*, Curcumin, Chitosan nanoparticles, Oxidative stress, TNF-α, Histopathological lesions, Drug development, Experimental models of disease

## Abstract

*Toxoplasma gondii* infection remains a significant global health concern, promoting the urgent need for effective therapeutic strategies. This study aimed to evaluate the therapeutic potential of chitosan nanoparticles (CSNPs) and curcumin-loaded chitosan nanoparticles (Cur-CSNPs) against the chronic *Toxoplasma gondii* (ME49 strain) in an experimental mouse model. This achieved by investigating their ability to reduce parasitic load, oxidative stress, histopathological lesion, and to enhance the host immune response. Sixty female BALB/c mice were divided into five groups: infected untreated group, Spiramycin®-treated group, CSNPs-treated group, Cur-CSNPs-treated group, and negative control group. The Cur-CSNPs-treated group exhibited the lowest brain cyst counts, along with significant reductions in cyst size. Hematological indices revealed no significant reduction in total white blood cell (WBC) counts or in the percentage of neutrophils, monocytes, and eosinophils in both the CSNPs and Cur-CSNPs treated groups, compared to the infected untreated group and Spiramycin-treated group. However, both nanoparticle-treated groups exhibited a significant decrease in the percentage of lymphocytes compared to the infected untreated group. Significant differences in total antioxidant capacity (TAC) and malondialdehyde (MDA) levels were observed, with the Cur-CSNPs treated group displaying values comparable to the negative control. Histopathological examination revealed substantial improvements in the brain, liver, and spleen tissues of Cur-CSNPs-treated animals, characterized by preserved tissue architecture and reduced inflammatory lesions. Immunohistochemical analysis further revealed reduced expression of IL-6 and TNF-α, indicating a mitigated inflammatory response. These findings highlight the promising therapeutic role of Cur-CSNPs in controlling chronic *T. gondii* infection and suggest their potential as a novel strategy for developing effective antiparasitic treatments.

**Supplementary Information:**

The online version contains supplementary material available at 10.1038/s41598-025-25252-5.

## Introduction

*Toxoplasma gondii* is a globally prevalent zoonotic protozoan parasite, infecting approximately one-third of the world’s vertebrate population, including both humans and animals^1^. Clinically, *T. gondii* exists in three forms: oocysts, shed exclusively by members of the Felidae family; rapidly proliferating tachyzoites, responsible for acute infection; and dormant bradyzoites, enclosed within tissue cysts^2^. Transmission occurs through ingestion of tissue cysts or oocysts present in contaminated food, water, or soil, as well as via transplacental (congenital) routes^3^. The public health impact of *T. gondii* is significant, particularly among pregnant women and immunocompromised individuals, for whom infection may result in severe complications, such as abortion, encephalitis, and neonatal mortality^4^. In countries such as Egypt, congenital toxoplasmosis continues to pose a major public health concern^5^.

*T. gondii* cysts exhibit prolonged persistence in diverse tissues, notably within the brain, thereby contributing to the risk of sustained active infection^6^. While current treatment options, such as Spiramycin®, offer some efficacy, they are limited by poor penetration across the blood–brain barrier (BBB) and potential side effects^7^. Natural therapeutic agents have shown promise; however, their clinical utility is often restricted by poor bioavailability and limited BBB permeability^8^. Nanotechnology presents a promising solution by enhancing the delivery of therapeutic compounds across the BBB and directly targeting brain-resident cysts^9^. This strategy also offers the potential to reduce systemic toxicity and improve drug tolerability^10,11^. Among the natural compounds under investigation, curcumin and chitosan have attracted particular interest due to their antimicrobial, antiparasitic, antioxidant, and immunomodulatory properties. When formulated as nanoparticles, these agents exhibit improved bioavailability, targeted delivery, and enhanced therapeutic outcomes^10^.

Curcumin, a polyphenol derived from *Curcuma longa*, demonstrates broad-spectrum activity against protozoan parasites, such as *Besnoitia besnoiti* and *Eimeria* spp., reducing oocyst shedding and improving host health^12^. Curcuminoids are recognized as “Generally Recognized as Safe” (GRAS) by the FDA, with good tolerability at doses up to 12,000 mg/day^13,14^. Its mechanisms of action include disruption of parasite membranes, inhibition of key enzymes, suppression of inflammatory mediators (e.g., TNF-α, IL-6, NF-κB), and induction of apoptosis in parasitic cells^15^. Nevertheless, its clinical application is limited by poor solubility and rapid metabolism. Nanoencapsulation using carriers, such as chitosan, can overcome these limitations by enhancing curcumin’s solubility and stability, as well as promoting its transport across the BBB^16^. Chitosan, a biodegradable polysaccharide derived from chitin, possesses intrinsic antimicrobial properties by interacting with negatively charged microbial membranes, increasing their permeability, and inducing cell death^17^. Additionally, chitosan modulates immune responses by stimulating macrophages and dendritic cells and enhancing cytokine production, thereby strengthening the host’s defense against intracellular pathogens, such as *T. gondii*^18^.

Oxidative stress is a key pathological feature of *T. gondii* infection, where an imbalance between reactive oxygen species (ROS) and antioxidants results in tissue injury and inflammation^19^. Curcumin mitigates oxidative stress by enhancing total antioxidant capacity (TAC) and reducing levels of malondialdehyde (MDA), a marker of lipid peroxidation^20^. TAC reflects the overall ability of body fluids to neutralize ROS, whereas elevated MDA levels indicate oxidative damage to cellular membranes^21,22^. Curcumin-loaded chitosan nanoparticles (Cur-CSNPs) offer a dual advantage: improved delivery of bioactive agents to infected tissues and direct antioxidant effects through ROS scavenging, lipid peroxidation inhibition, and enhancement of endogenous antioxidant defenses^23^. Therefore, this study aims to evaluate the therapeutic potential of curcumin-loaded chitosan nanoparticles (Cur-CSNPs) and chitosan nanoparticles (CSNPs) in the treatment of cerebral toxoplasmosis. The investigation includes quantification of brain bradyzoites, histopathological and immunohistochemical analyses of brain, spleen, and liver tissues, as well as assessment of oxidative stress biomarkers (TAC) and (MDA); to determine the efficacy and protective effects of the proposed nanotherapeutics.

## Materials and methods

### Ethics statement

The experimental procedures of the present study strictly adhered to national and international ethical guidelines. Approval for all procedures was obtained from the Faculty of Veterinary Medicine, Assiut University (Approve number: 06/2023/0058), as sanctioned by its Research Ethical Committee. The study is reported in accordance with the ARRIVE guidelines (https://arriveguidelines.org)^24^.

### Parasite

*T. gondii* Bradyzoites from the ME49 (cyst-forming type II) strain were provided by the Schistosome Biological Supply Program (SBSP), Theodor Bilharz Research Institute (TBRI), Egypt. Brains of the infected mice were extracted and homogenized in saline. Cysts were counted microscopically in 25 µL of brain suspension^25^.

### Genetic confirmation of *Toxoplasma* strain

Brain tissue samples were used for genomic DNA extraction with the QIAamp DNA Mini Kit (Qiagen, Hilden, Germany, Cat. No. 51306). A 196-bp fragment of the B1 gene DNA barcode region was amplified using specific forward (5′-GGAACTGCATCCGTTCATGAG-3′) and reverse (5′-TCTTTAAAGCGTTCGTGGTC -3′) primers^26^. The PCR Master Mix was prepared using EmeraldAmp® GT PCR Master Mix (Takara Bio Inc., Shiga, Japan, Cat. No. RR310A), containing 12.5 μl of 2 × premix, 5.5 μl of PCR-grade water, 1 μl each of forward and reverse primers (20 pmol each), and 5 μl of template DNA, resulting in a total reaction volume of 25 μl. Amplification of the B1 gene involved an initial denaturation at 95 °C for 5 min, followed by 35 cycles of denaturation at 94 °C for 30 s, annealing at 60 °C for 30 s, and extension at 72 °C for 30 s, with a final extension step at 72 °C for 7 min. Post-amplification, PCR products were subjected to electrophoresis on a 1.5% agarose gel using horizontal cell (Compact M, Biometric, Germany) and visualized using ultraviolet illumination to confirm successful amplification^27^.

The amplified products were purified using the QIAquick PCR Purification Kit (Qiagen Inc., Valencia, CA, USA, Cat. No. 28104), following the manufacturer’s instructions. The process involved binding DNA to a silica membrane in a spin column, washing, and eluting with Buffer EB. Purification typically took approximately 10 min, yielding PCR products suitable for further applications. The purified PCR products were sequenced in both directions using an automated DNA sequencer (ABI 3130 Genetic Analyzer, Applied Biosystems, Foster City, CA, USA). Sequencing reactions were performed using the BigDye® Terminator v3.1 Cycle Sequencing Kit (Applied Biosystems/PerkinElmer, Foster City, CA, USA).

### Phylogenetic analysis

Sequences obtained were compared with existing sequences in GenBank using the NCBI BLAST program^28^. Phylogenetic analysis was performed using the CLUSTAL W multiple sequence alignment program (version 12.1 of the MegAlign module)^29^. Phylogenetic trees were constructed in MEGA11 using maximum likelihood, neighbor-joining, and maximum parsimony methods^30^.

### Preparation of chitosan nanoparticles

Chitosan nanoparticles (CSNPs) were prepared using an ionotropic gelation process, according to Calvo et al.^31^. Briefly, a solution of chitosan (0.75 mg/ml) and tripolyphosphate (TPP, 1.5 mg/mL) as a crosslinking agent was prepared in 0.1 M acetate buffer (pH 5.3). A volume of 1.5 mL of TTP solution (TPP-chitosan ratio = 0.3) was rapidly added under magnetic stirring (500 rpm) to 10 mL of the chitosan colloid solution. The mixture was stirred for 15 min at room temperature.

### Preparation of curcumin-loaded chitosan nanoparticles (Cur-CSNPs)

Curcumin-loaded nanoparticles was prepared according to Yadav et al.^32^. Briefly, 20 mL of a 0.3% chitosan solution in diluted acetic acid (35 mM) was mixed with 0.5 mL of Tween 80 and stirred continuously stirred for 1 h. Then, 250 μL of curcumin solution (13.75 mg/ml in chloroform) was added in 20 μL aliquots under stirring. Residual chloroform was removed from the nanoparticle suspension through a three-step process: evaporation at room temperature under stirring for 4 h to eliminate the bulk solvent; centrifugation to separate unreacted or insoluble materials that could entrap chloroform; and repeated washing with a chloroform-free medium (distilled water or buffer) via centrifugation to ensure complete removal of residual solvent^30^. After 1 h, 0.5 ml of 20% sodium sulfate solution was added drop by drop, and the mixture was stirred for an additional 30 min. Then, 0.1 mL of glutaraldehyde was added to the mixture and stirred for an additional 30 min; to crosslink the nanoparticles. Subsequently, 1 ml of 10% sodium meta-bisulfite was added, and the mixture was stirred for another 30 min. The solution was allowed to stand for 12 h, then dialyzed twice against water for 24 h at 4 °C and twice against normal saline for 24 h at 4 °C. The dialyzed solution was kept at 4 °C^10^.

### Encapsulation efficiency (EE%) and drug loading (DL) of the Cur-CSNPs

The encapsulation efficiency of the prepared Cur-CSNPs was determined using an indirect method^10^. Briefly, freshly prepared Cur-CSNPs were centrifuged using Amicon® Ultra-15 centrifugal filters (100 kDa NMWL, Merk Millipore, Darmstadt, Germany) at 6000 rpm for 30 min. The filtrate was analyzed using a Shimadzu UV–Vis spectrophotometer (model UV-1601 PC, Kyoto, Japan) at λ = 450 nm. Drug concentration (DL) was calculated from calibration curves and encapsulation efficiency was determined using the following Equation:^33^

$${\mathrm{Encapsulation}}\;{\mathrm{efficiency}}\;\left( \% \right) = \left[ {\frac{{\left( {{\mathrm{Total}}\;{\mathrm{drug}}\;{\mathrm{concentration}} - {\mathrm{drug}}\;{\mathrm{concentration}}\;{\mathrm{in}}\;{\mathrm{filtrate}}} \right)}}{{\text{Total drug concentration}}}} \right] \times {1}00.$$

Drug loading was calculated as:

$${\mathrm{Drug}}\;{\mathrm{loading}}\;\left( \% \right) = \left( {\frac{{\left( {{\mathrm{Total}}\;{\mathrm{drug}}\;{\mathrm{added}} - {\mathrm{Free}}\;{\mathrm{drug}}} \right)}}{{\left( {{\mathrm{Total}}\;{\mathrm{drug}}\;{\text{added }} - {\text{ Free}}\;{\text{drug }} + {\text{ Carrier}}\;{\mathrm{polymer}}\;{\mathrm{weight}}} \right)}}} \right) \times \;{1}00.$$

### Characterization of CSNPs and Cur-CSNPs

The physical properties and morphology of chitosan nanoparticles (CSNPs) and curcumin-loaded chitosan nanoparticles (Cur-CSNPs) were visualized using JOEL, JSM-5400LV scanning electron microscope (SEM) and JEOL 100 CX II TEM (Tokyo, Japan) at the Electron Microscope Unit, Assiut University, Egypt^10^. Particle size and size distribution were determined using dynamic light scattering (DLS), and zeta potential was measured with a Zetasizer Nano ZS (Malvern Panalytical Ltd., Malvern, UK).

### Reference drug

Spiramycin® tablets (Rovamycin® -Sanofi Aventis Pharmaceutical Company, France) were weighed and crushed into powder. The dosage was then calculated for each mouse and dissolved in 100 µl of distilled water for oral administration^34^.

### Experimental design

Sixty female BALB/c mice (7–8 weeks old, 20–25 g) were obtained from the Schistosome Biological Supply Program (SBSP) at Theodor Bilharz Research Institute (TBRI). Animals were housed at the animal facility of Assiut University, Egypt, under controlled conditions (temperature: 24 ± 2 °C; 12-h light/dark cycle; relative humidity: 40–70%). Throughout the study, mice were maintained under specific pathogen-free conditions with ad libitum access to tap water and a standard diet (7% simple sugars, 3% fat, 50% polysaccharide, 15% protein, and energy 3.5 kcal/g)^10^. The mice were divided into five experimental groups as follows:

*Control negative group* Uninfected and untreated, serving as the baseline reference.

*The infected untreated group* Each mouse was orally inoculated with 250 µL of brain suspensions containing 10 cysts. This inoculation method consistently established chronic infection without mortality^25^.

*Spiramycin-treated group* Each mouse was orally administered with 400 mg/kg/day of Rovamycin®^35^ dissolved in 100 µL of normal saline.

*CSNPs-treated group* Each mouse was orally administered with 30 mg/kg/mouse/day of chitosan nanoparticles^35^ dissolved in 100 µL of normal saline.

*Cur-CSNPs-treated group* Each mouse was orally administered with 0.5 mL of the Cur-CSNPs formulation, corresponding to approximately 6.875 mg of curcumin, as determined by the pilot study (supplementary file, Table S1and Figure S1).

After 60 days post-infection, mice were treated daily for 10 days by oral gavage. The animals from all groups were euthanized at the end of day 70^th^ post-infection. Mice were anesthetized via intraperitoneal injection of sodium thiopental (100 mg/kg) and subsequently euthanized by cervical dislocation to ensure death^36^.

### Parasitological analysis

The entire brain, liver, and spleen from six animals/group were removed, weighed, and homogenized in 2 mL of sterile phosphate buffer saline (PBS). Tissue impression smears were stained with 10% Giemsa solution (Merck, Kenilworth, NJ, USA) in PBS. Morphological changes in bradyzoites in the infected and treated groups were examined using an oil immersion lens (ten fields per slide, three slides per organ). The mean size and number of bradyzoites were measured using a light microscope (Olympus BX43F, Tokyo163-0914, Japan) equipped with a camera (Olympus, EP50, Tokyo, Japan) at the Department of Parasitology, Faculty of Veterinary Medicine, Assiut University^37^.

The percentage reduction in the parasite count was calculated as follows: %R = 100(C − E)/C.

Where %R is the percentage of reduction, C is the mean count of cysts in the infected untreated positive control group, and E is the mean cyst count in an experimental group^36^.

### Blood collection and hematological analysis

At the end of the experiment, whole blood samples (12 per group) were collected. Six blood samples per group were collected from the retro-orbital sinuses into sterile vacuum tubes containing EDTA as an anticoagulant for haematological analysis, including differential leukocyte counts, using a Veterinary Exigo Hematology Analyzer^38^. Another six blood samples per group were collected into plain sterile tubes for serum analysis. The samples were allowed to clot, and sera were separated by centrifugation (PCL-012, Gemmy industrial corp., Taiwan) at 3000 rpm for 20 min and stored at −80 °C until further biochemical analysis^10^.

### Oxidative stress biomarkers

Serum levels of malondialdehyde (MDA) and total antioxidant capacity (TAC) were analyzed using colorimetric assay kits provided by Bio Diagnostics, Egypt according to Ohkawa et al.^39^ and Koracevic et al.^40^, respectively. Biochemical analyses were utilized using a 6705 UV/Vis Spectrophotometer (JENWAY) spectrophotometer at the Central Laboratory of the Pathology Department, Faculty of Veterinary Medicine, Assiut University, Assiut, Egypt.

### Histopathological examination

Brain, liver, and spleen tissues from six animals per group were processed for histopathological and immunohistochemical analysis. Tissue specimens were fixed in 10% neutral buffered formalin and dehydrated in ascending concentrations of ethyl alcohol series. The tissue specimens were cleared in xylene for 24 h and then embedded in paraffin and cut into 4 μm sections. The resulting sections were stained with hematoxylin and eosin and examined using a light microscope (Olympus CX31, Tokyo, Japan) and captured by a camera (Toup view, LCMos10000KPA, China) at the Department of Pathology, Faculty of Veterinary Medicine, Assiut University^41^. Histopathological lesions were scored semi-quantitatively by examining 10 random fields per section per organ (three sections per organ for six different animals per group) on a fixed high-power field at × 400 using a light microscope ^42^.

### Immunostaining and quantitative analysis of IL-6 and TNF-α

Immunohistochemical staining for IL-6 and TNF-α was underwent on brain, liver, and spleen tissues from six mice per group. Paraffin-embedded tissues were mounted on electrostatically charged slides, deparaffinized in xylene, rehydrated in series alcohols (100% I and II, 90% I and II, 15 min each), and washed in phosphate buffer saline (PBS, pH 7.4) three times for 5 min each. Slides were treated with heat-induced antigen retrieval (in citrate buffer at 95 ºC for 20 min), hydrogen peroxide (3%)-mediated inhibition of endogenous peroxidase activities (at room temperature for 10 min), and 5% bovine serum albumin-mediated inhibition of the nonspecific bindings (at room temperature for 10 min). The sections were allowed to cool at room temperature for 30 min and washed with PBS for 15 min. Then, the sections were incubated overnight at 4°C with anti-IL-6 (diluted 1:50, A0286) and anti- TNF-α (diluted 1:100, A11534) rabbit polyclonal primary antibodies (AB clonal Company, 500W Cummings Park, Ste. 6500, Woburn, MA 01,801, USA). Slides were then incubated with Econo Tek biotinylated anti-polyvalent secondary antibody and Econo Tek HRP conjugate for 30 min each at room temperature. The slides were washed with PBS (pH 7.4) for 5 min and treated with 3,3'-diaminobenzidine chromogen and its substrate for 5–10 min at room temperature. The slides were then counterstained with Harris hematoxylin for 30 s, dehydrated, cleared in xylene, and mounted using DPX. Negative controls did not include the use of primary antibodies during the staining process according to Hamad, et al.^43^.

The area percentage of IL-6 and TNF-α immunostaining was identified using digital image analysis (Image J software). The proportion of positively stained cells was expressed as a percentage of total pixels in the optical view. The calculation process was performed on ten fixed high-power fields (× 400) using a light microscope (Olympus CX31, Tokyo, Japan) supported with a digital camera (Toup view, LCMos10000KPA, China). Ultimately, the area percentage of immunolabeling in all experimental groups was presented as means, which were statistically analyzed^44,45^.

### Statistical analysis

Data analysis was performed using R statistical software (version 4.2.2). One-way analysis of variance (ANOVA) was used to assess statistical significance among experimental groups for each variable. When significant differences were detected, a post-hoc Duncan multiple range test was conducted to identify specific group differences. Data were expressed as mean ± SEM. *P* values less than 0.05 were considered statistically significant. Additionally, graphical representations of the data were generated using the plot package in the R program.

## Results

### Characterization of nanoparticles

**Fig. 1:**
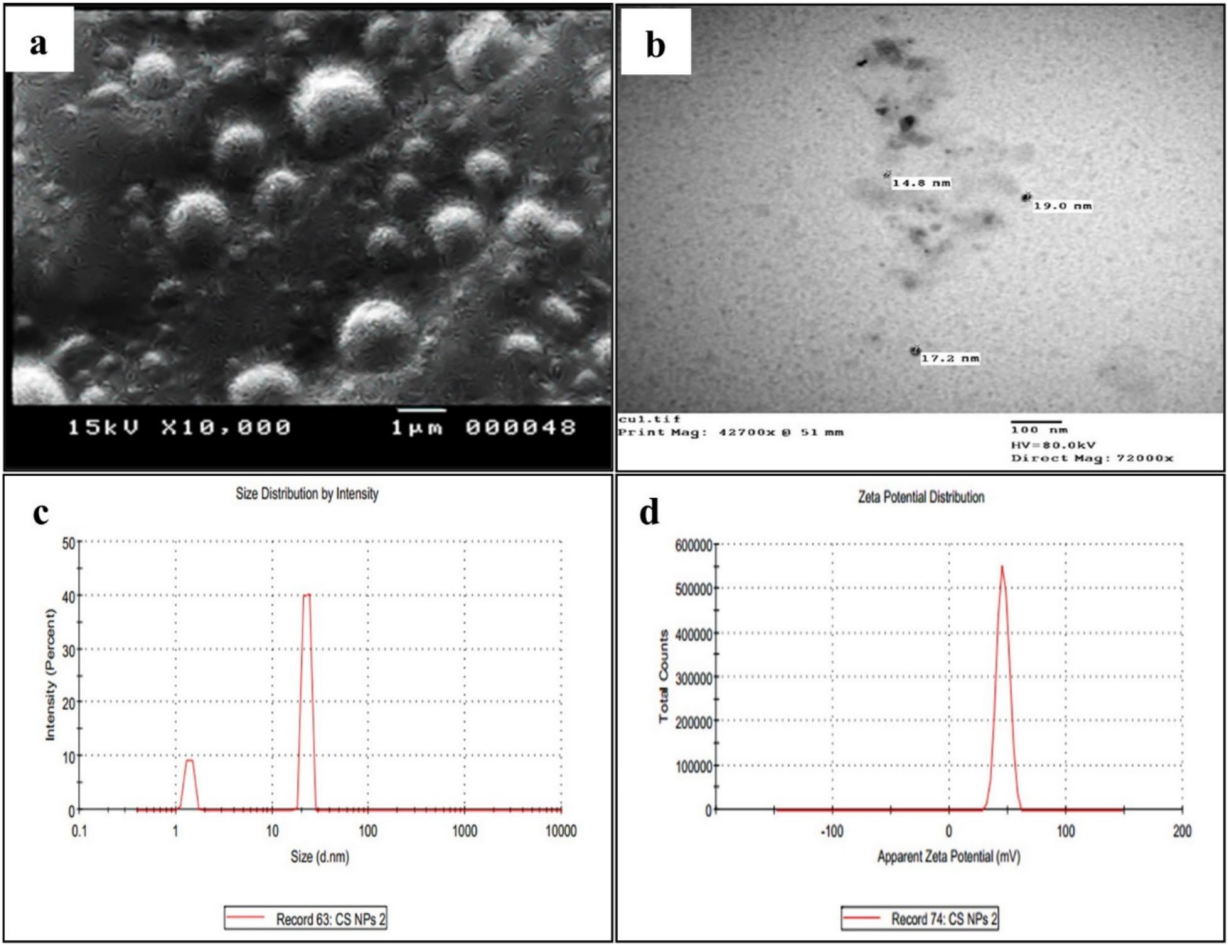
Characterization of chitosan nanoparticles showing (**a**) SEM images of CSNPs at 10,000 × magnification; (**b**) TEM images of CSNPs at a magnification of (42700x; scale bar = 100 nm) shows the internal structure of nanoparticles; (**c**) DLS analysis reported two main peaks for CSNPs particle size in nano range: 22.68 nm and 1.391 nm; (**d**) Zeta potential and PDI were 45.8 mV and 1.0 respectively.

**Fig. 2:**
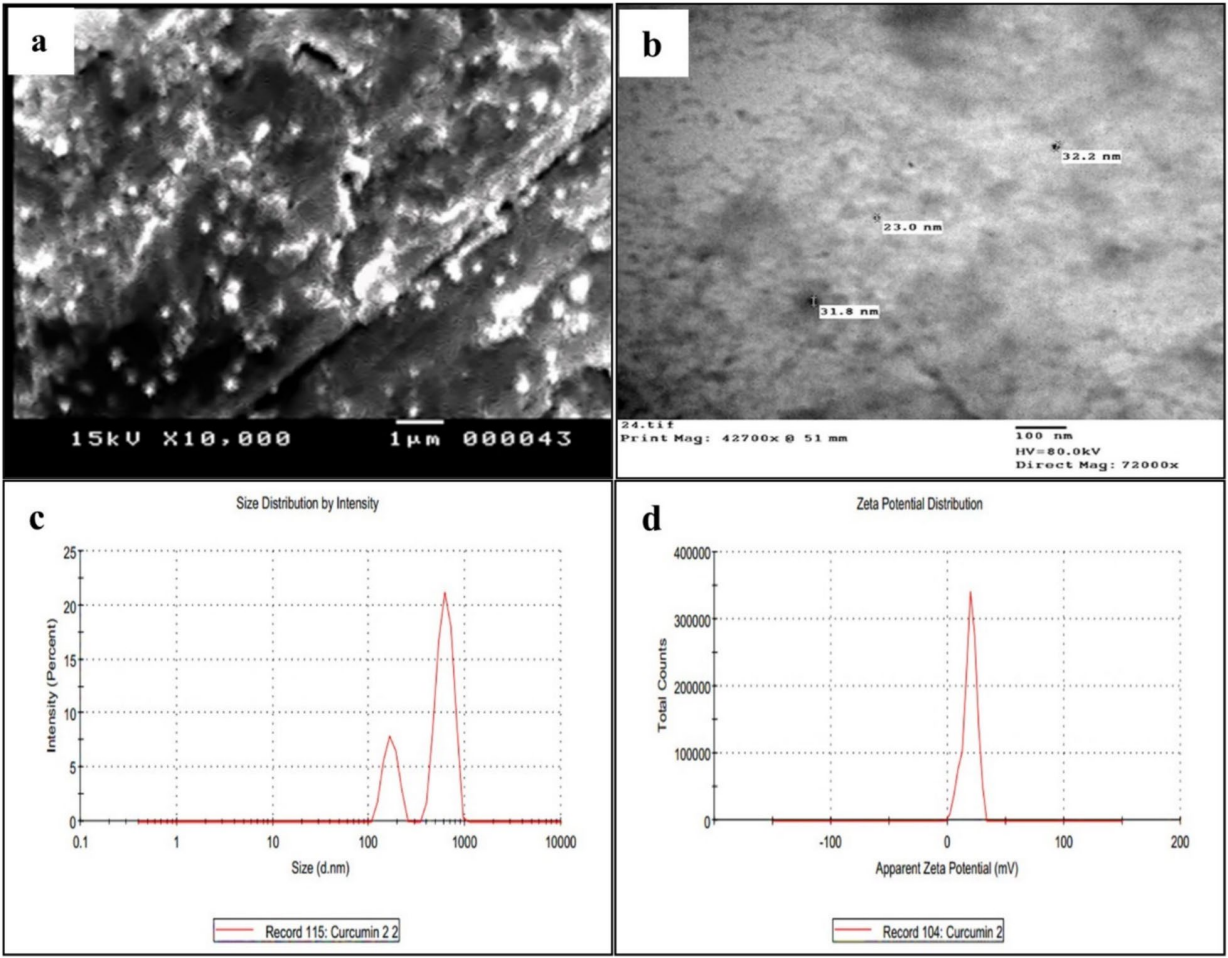
Characterization of curcumin-loaded chitosan nanoparticles showing (**a**) SEM images of Cur-CSNPs at 10,000 × magnification; (**b**) TEM images of Cur-CSNPs at a magnification of (42700x; scale bar = 100 nm) shows the internal structure of nanoparticles; (**c**) DLS analysis reported two main peaks for Cur-CSNPs particle size in nano range: 623.1 nm and 169.6 nm; (**d**) Zeta potential and PDI were19.0 mV and 0.589, respectively.

Scanning electron microscopy of CSNPs and Cur-CSNPs revealed a smooth, spherical shape with distinct and stable margins (Figs.1a and 2a). Transmission electron microscopy confirmed the size and shape of CSNPs, demonstrating a predominantly regular and spherical morphology (Fig. 1b). Whereas Cur-CSNPs displayed regular shapes with predominantly spherical nanoparticles (Fig. 2b ).

Dynamic light scattering analysis (DLS) of CSNPs revealed two main peaks in the nano range at 1.391 nm and 22.68 nm (Fig. 1c). The characterization of nanoparticles revealed that plain CSNPs exhibited a mean particle size of 80.4 nm with a moderate polydispersity index (PDI = 0.57) and a high positive zeta potential of + 45.8 mV (Table 1, Fig. 1c and d). In contrast, Cur-CSNPs revealed two main peaks in the nano range at 169.6 nm and 623.1 nm, with an increase in mean size to 307.2 nm and a high PDI of 0.94 (Table 1, Fig. 2c). The zeta potential decreased to + 19.0 mV (Table 1, Fig. 2 d).

**Table 1:** Physicochemical Properties of Chitosan Nanoparticles (CSNPs) and Curcumin-Loaded Chitosan Nanoparticles (Cur-CSNPs).

Sample	Particle Size (nm, Z-Average)	Zeta potential (mV)	PDI
CSNPs	221.1	+ 45.8 (± 5.23)	0.57
Cur-CSNPs	307.2	−19.0 (± 5.88)	0.93

Chitosan nanoparticles (CSNPs), curcumin-loaded CSNPs (Cur-CSNPs), polydispersity index (PDI). Data of Zeta potential is presented as mean ± standard deviation.

### Encapsulation efficiency of Cur-CSNPs

The initial drug concentration was 0.1535 mg/mL, with an absorbance of 0.325 for the supernatant. The free drug concentration was calculated as 0.00672 mg/mL. The efficiency of encapsulation was determined to be 95.6%. The loading capacity (LC) of the nanoparticles was calculated to be 4.65%, reflecting the proportion of curcumin relative to the total nanoparticle mass.

### Molecular analysis for *Toxoplasma* species

The B1 gene of *T. gondii* DNA was amplified via polymerase chain reaction (PCR), resulting in a clear band at 196 bp (Supplementary File, Figure S2). Subsequent sequencing of the amplified products confirmed *Toxoplasma* sequences, which were compared with GenBank sequences in NCBI. These sequences showed a high identity of 98–100% with *T. gondii* sequences. The sequence generated in this study was deposited in GenBank under accession number OP991839 for future reference and accessibility. Phylogenetic relationship between the present strain and 26 reference *T. gondii* strains revealed distinct genetic distinctions among them (Supplementary File, Figure S3).

### Parasite count and reduction percentage (%R)

**Fig. 3:**
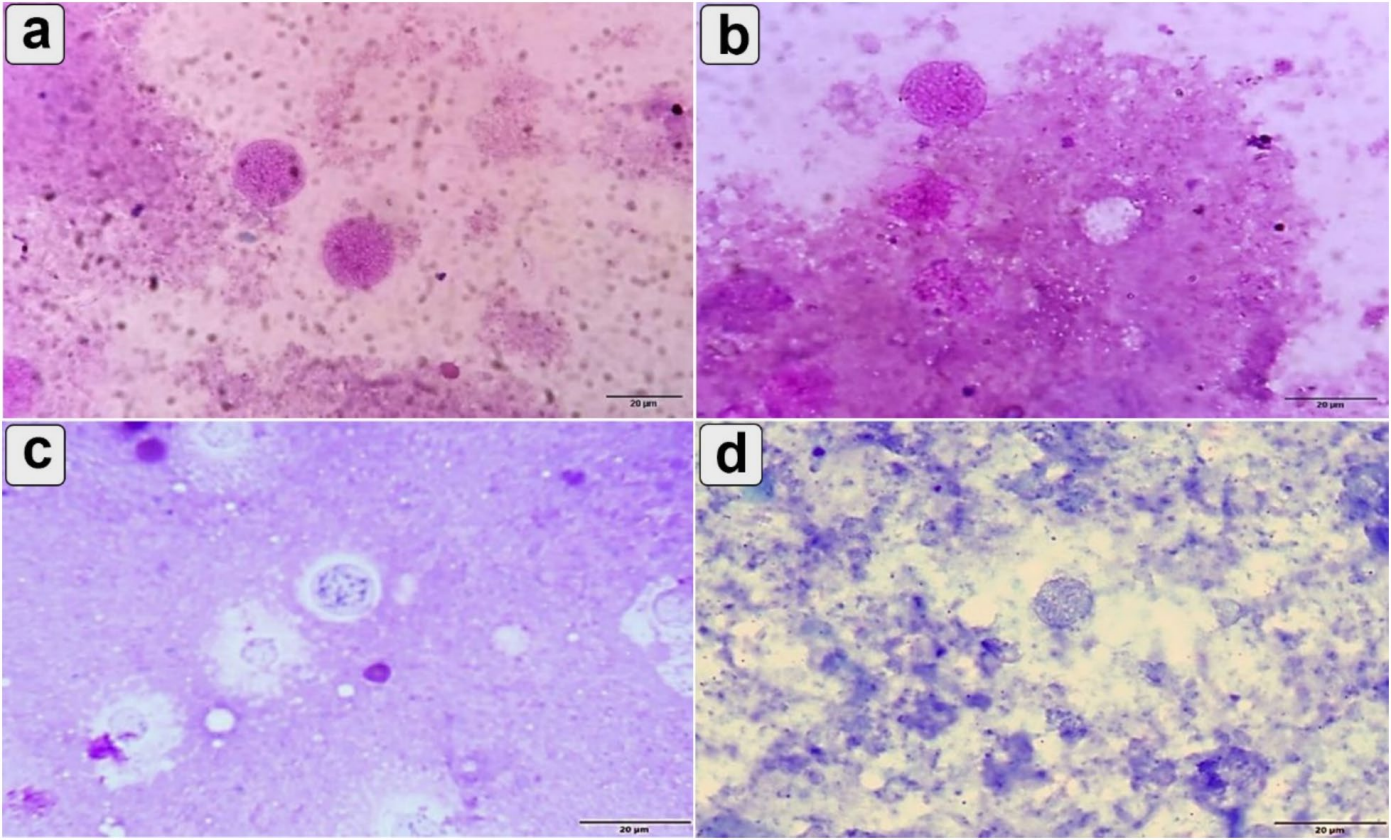
Bradyzoite Stages of *Toxoplasma gondii* in mouse brain stained with Giemsa in different treatment groups (n = 6 samples/group); scale bar = 20μm. (**a**) Bradyzoite in the infected-untreated group. (**b**) Bradyzoite in the Spiramycin treated group. (**c**) Bradyzoite in the CSNPs treated group. (**d**) Bradyzoites in the Cur-CSNPs treated group exhibited a significant reduction in cyst size (*P* = 0.001) compared to other treated groups, whereas no statistically significant difference was observed between other groups (*P* > 0.05).

The infected untreated group and spiramycin®-treated groups showed large, regular, intact *T. gondii* cysts with less damaged (Fig. 3 a and b). However, cyst walls exhibited signs of damage or disruption in the CSNPs and Cur-CSNPs-treated groups (Fig. 3 c and d).

As shown in Table 2, the Cur-CSNPs-treated group had the smallest cysts (Fig. 1d) with a significant reduction in cyst size (*P* = 0.001) compared to other treated groups. No statistically significant differences in cyst size were observed among the other groups (*P* > 0.05).

**Table 2:** Mean size and number of brain cysts of T. gondii in mice across different treatment groups (n = 6 samples/group).

Cyst	Groups				*P*-Value
	Infected-untreated	Spiramycin®	CSNPs	Cur-CSNPs	
Size	7.52^a^ ± 0.18	7.57^a^ ± 0.21	7.32^a^ ± 0.22	5.96^b^ ± 0.31	0.001
Count	6.00^a^ ± 0.58	5.00^ab^ ± 0.58	4.33^ab^ ± 0.33	3.00^b^ ± 1.15	0.041
Reduction %	–	16.7%	27.8%	50%	–

^a,b^ indicates significant difference between groups (*P* < 0.05). CSNPs (chitosan nanoparticles), Cur-CSNPs (curcumin-loaded on chitosan nanoparticles). ‟–” no cysts detected.

Furthermore, cyst counts were significantly increased (*P* = 0.041) in the infected untreated group compared to the treated groups. However, no differences were observed in the number of tissue cysts among the treated groups. Notably, a high reduction in the number of cysts was recorded in the Cur-CSNPs-treated group.

To assess the efficacy of treatments, reduction percentages were calculated relative to the infected untreated group. The Cur-CSNPs-treated group showed the highest percentage of reduction (50%), followed by the CSNPs-treated group (27.8%), and Spiramycin-treated group (16.7%).

### Differential blood cell count

**Fig. 4:**
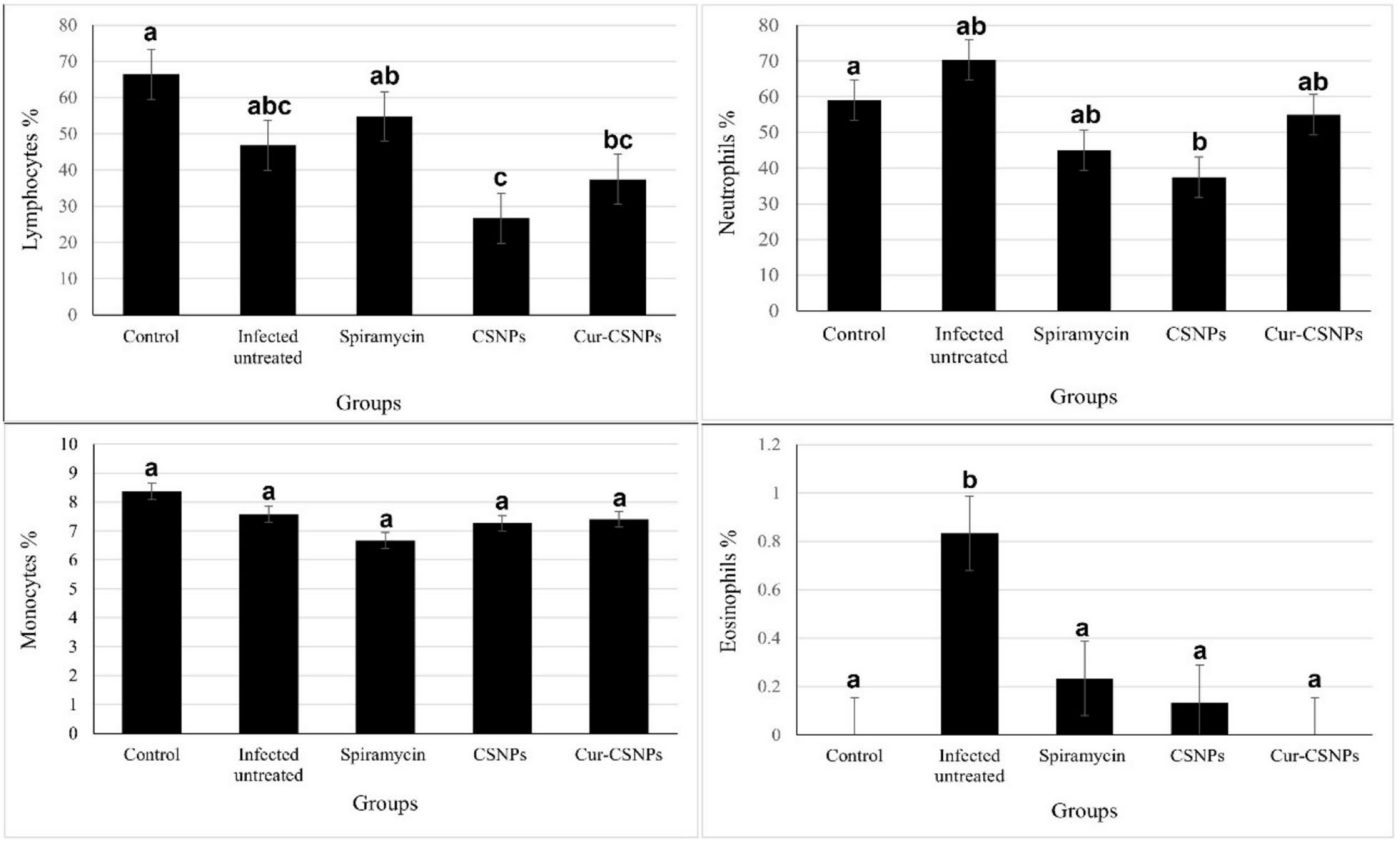
Differential blood cell count in the different groups (n = 6 samples/group). a,b,c,d indicate significant differences between groups (*P* < 0.05). Similar letters indicate non-significant differences (*P* > 0.05).

A non-significant decrease in total white blood cell (WBC) counts (*P* = 0.241) was observed in all treated groups (7.5 ± 1.1, 6.1 ± 2.5, and 7.2 ± 3 for Spiramycin, CSNPs, and Cur-CSNPs- treated groups, respectively) compared to the infected untreated (19.9 ± 9) group. However, there was a statistically significant decrease in the percentage of lymphocytes in the CSNPs and Cur-CNPs-treated groups compared to the infected untreated group and Spiramycin-treated group. Furthermore, all treated groups showed a non-significant decrease in the percentage of neutrophils (*P* = 0.103) and monocytes (*P* = 0.769) compared to the infected untreated group. Eosinophil percentage was significantly increased in the infected untreated group (*P* = 0.014) compared to the control and treated groups (Fig. 4).

### Biochemical analysis

**Fig. 5:**
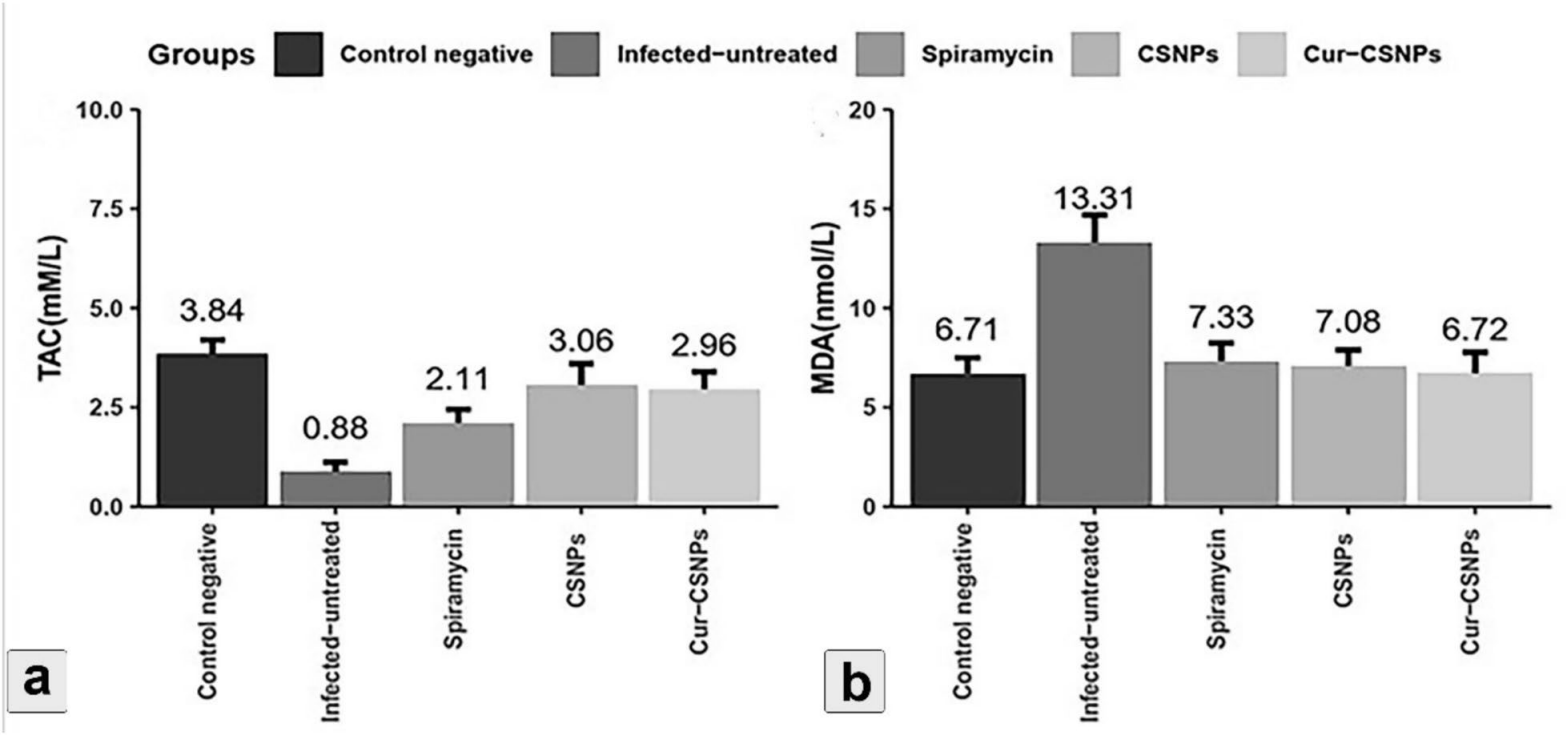
Differential effects of treatments on Total Antioxidant Capacity (TAC) and Malondialdehyde (MDA) levels across experimental groups (n = 6 samples/group). Data are presented as means ± SEM and were analyzed using one-way ANOVA (a post-hoc Duncan multiple range test). Mean values of TAC and MDA for each group are indicated above the bars. Statistical significance was considered at *P* < 0.05. CSNPs: Chitosan nanoparticles-treated group; Cur-CSNPs: curcumin-loaded Chitosan nanoparticles-treated group.

Statistical analysis revealed significant variations in TAC levels among groups. A significant reduction (*P* < 0.001) in the TAC level was observed in the infected untreated group and Spiramycin® treated groups compared to the negative control group. Whereas a significant increase (*P* < 0.001) was observed in the level of MDA in the infected untreated group compared to the treated groups. The CSNPs and Cur-CSNPs-treated groups exhibited no significant changes in TAC and MDA levels compared to the negative control group (Fig. 5).

### Histopathological findings

**Fig. 6:**
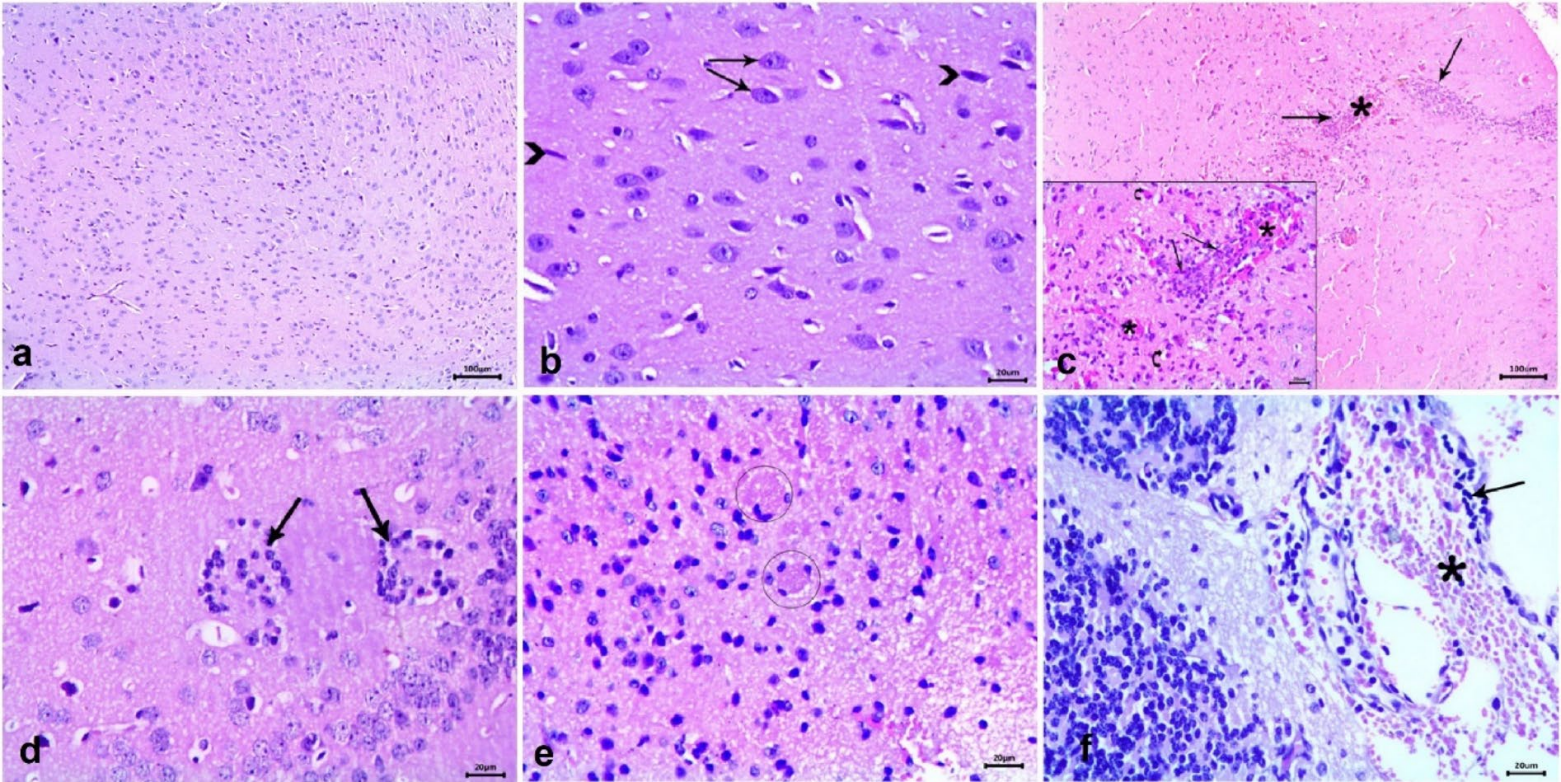
Photomicrograph of brain tissue sections (n = 6 samples/group) stained with hematoxylin and eosin from control and infected untreated groups. (**A**, **B**) Negative control demonstrate normal structure of brain tissue, comprising neurons (arrow) and glia cells (arrowhead). (**C**–**F**) Infected untreated group; (**C**) Congested blood vessel (asterisk), focal mononuclear cells infiltration (arrow), and neuronal degeneration (curved arrow). (**D**) gliosis (arrow). (**E**) Multiple tissue cysts of *T. gondii* with bradyzoites (circle). (**F**) Hemorrhage (asterisk) and mononuclear cells infiltration in meninges (arrow). (A and C, 10X) and (B, inset, D, E, and F, 40X).

**Fig. 7:**
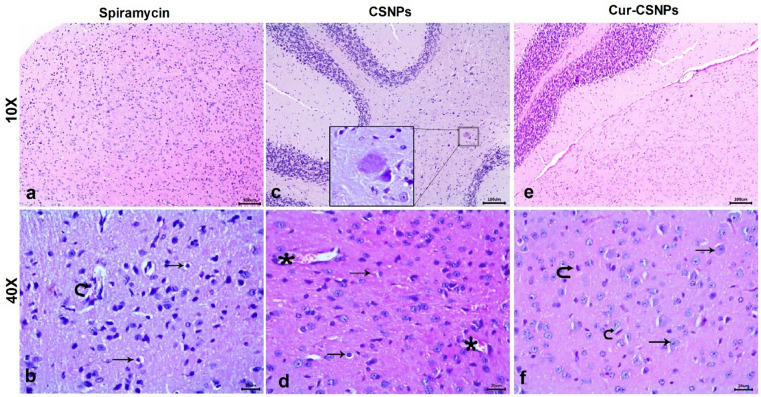
Photomicrograph of brain tissue sections (n = 6 samples/group) stained with hematoxylin and eosin from Spiramycin-treated group (**A**, **B**) demonstrating congested blood vessel (curved arrow), and neuronal degeneration (arrow), CSNPs-treated group (**C**, **D**) demonstrating tissue cyst (square), congested blood vessel (asterisk), and neuronal degeneration (arrow), inset is high magnification, and Cu-CSNPs-treated group (**E**, **F**) demonstrating apparently normal appearance of neurons (arrow) and glia cells (curved arrow). CSNPs: Chitosan nanoparticles-treated group; Cur-CSNPs: curcumin-loaded Chitosan nanoparticles-treated group.

**Fig. 8:**
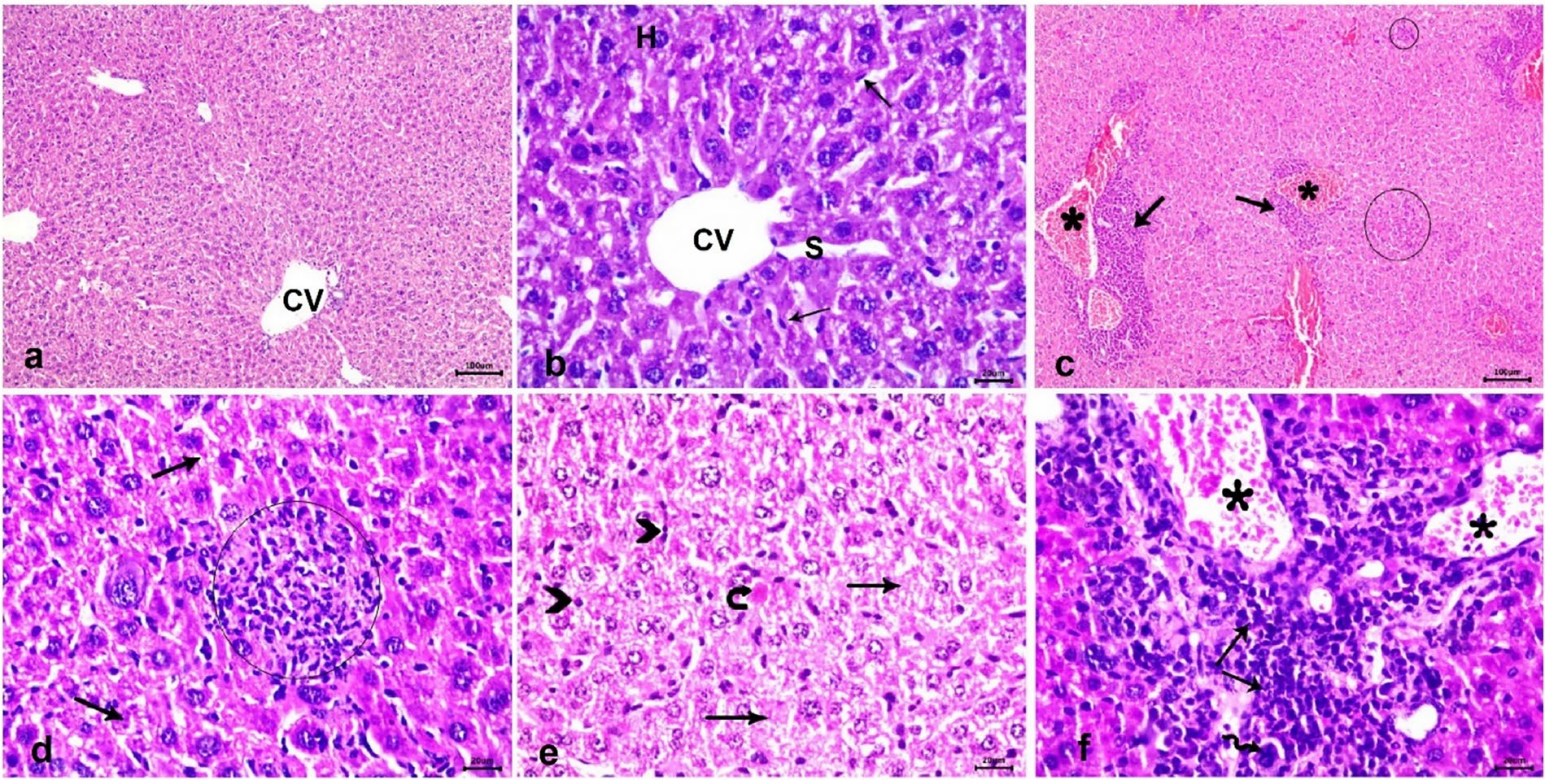
Photomicrograph of liver tissue sections (n = 6 samples/group) stained with hematoxylin and eosin from control and infected untreated groups. (**A**, **B**) Negative control demonstrate normal structure of hepatic parenchyma, comprising central vein (CV), cords of hepatocytes (H) with blood sinusoids in between (S), and Kupffer cells (arrow). (**C**–**F**) Infected untreated group; (**C**) Vascular congestion (asterisk), perivascular aggregations of mononuclear cells (arrow), and multifocal areas of coagulative necrosis infiltrated with mononuclear cells (circle). (**D**) Diffuse hepatocytes vacuolar degeneration (arrow) and focal area of coagulative necrosis infiltrated with inflammatory cells (circle). (**E**) coagulative necrosis of hepatocytes (arrow), tissue cyst (curved arrow), and Kupffer cell reaction (arrowhead). (**F**) Congestion of blood vessels (asterisk), tissue cyst (wavy arrow), and aggregates of mononuclear cells in portal area (arrow). (A and C, 10X) and (B, D, E, and F, 40X).

**Fig. 9:**
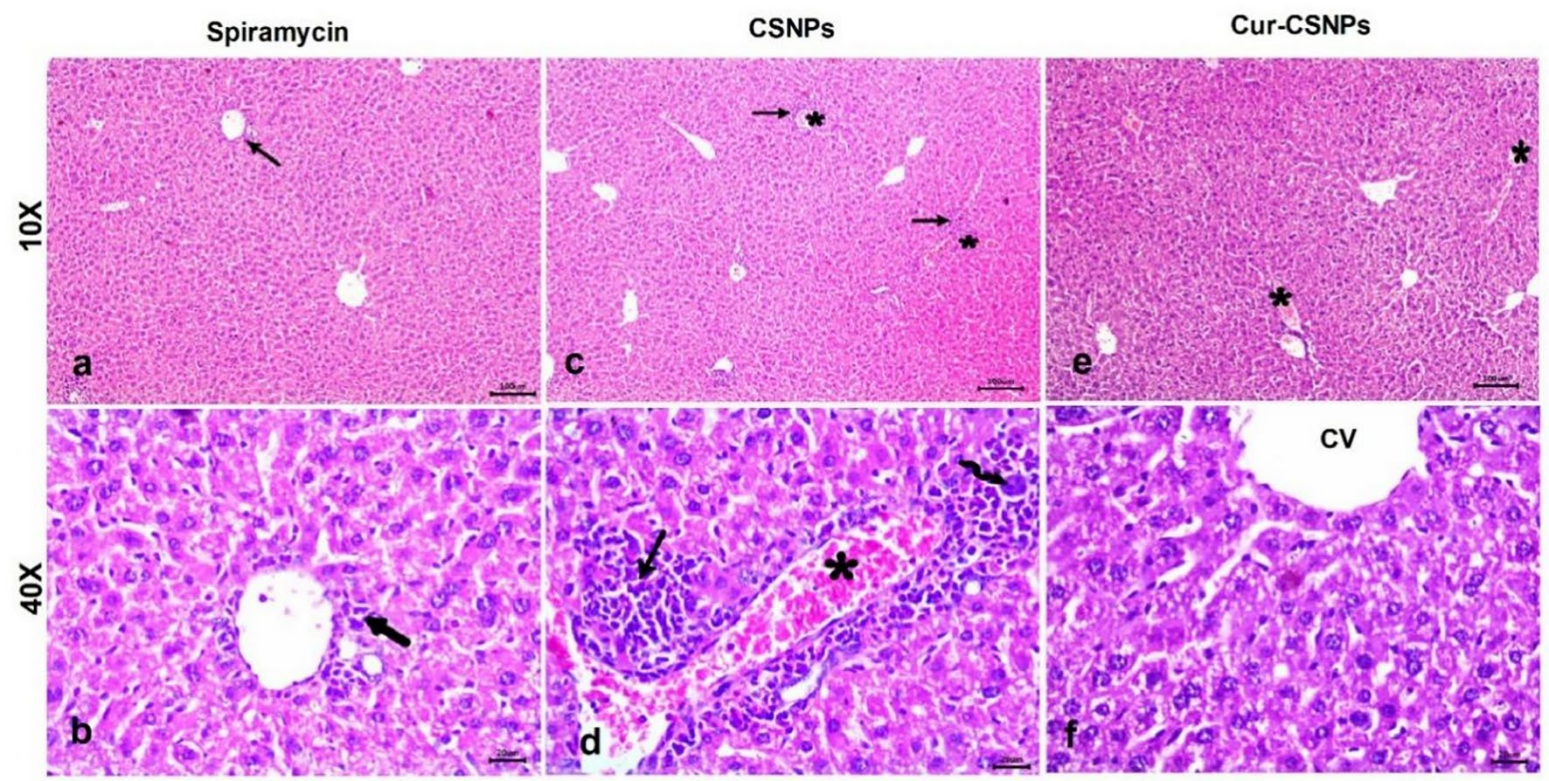
Photomicrograph of liver tissue sections (n = 6 samples/group) stained with hematoxylin and eosin from Spiramycin-treated group (**A**, **B**) demonstrating slight infiltration of mononuclear cells in portal areas (arrow), CSNPs-treated group (**C**, **D**) demonstrating tissue cysts (wavy arrow) and vascular congestion (asterisk) with perivascular inflammatory cells infiltration (arrow), and Cu-CSNPs-treated group (**E**, **F**) demonstrating normal structure of the hepatic parenchyma with mild vascular congestion (asterisk) in some examined lobules. CSNPs: Chitosan nanoparticles-treated group; Cur-CSNPs: curcumin-loaded Chitosan nanoparticles-treated group.

**Fig. 10:**
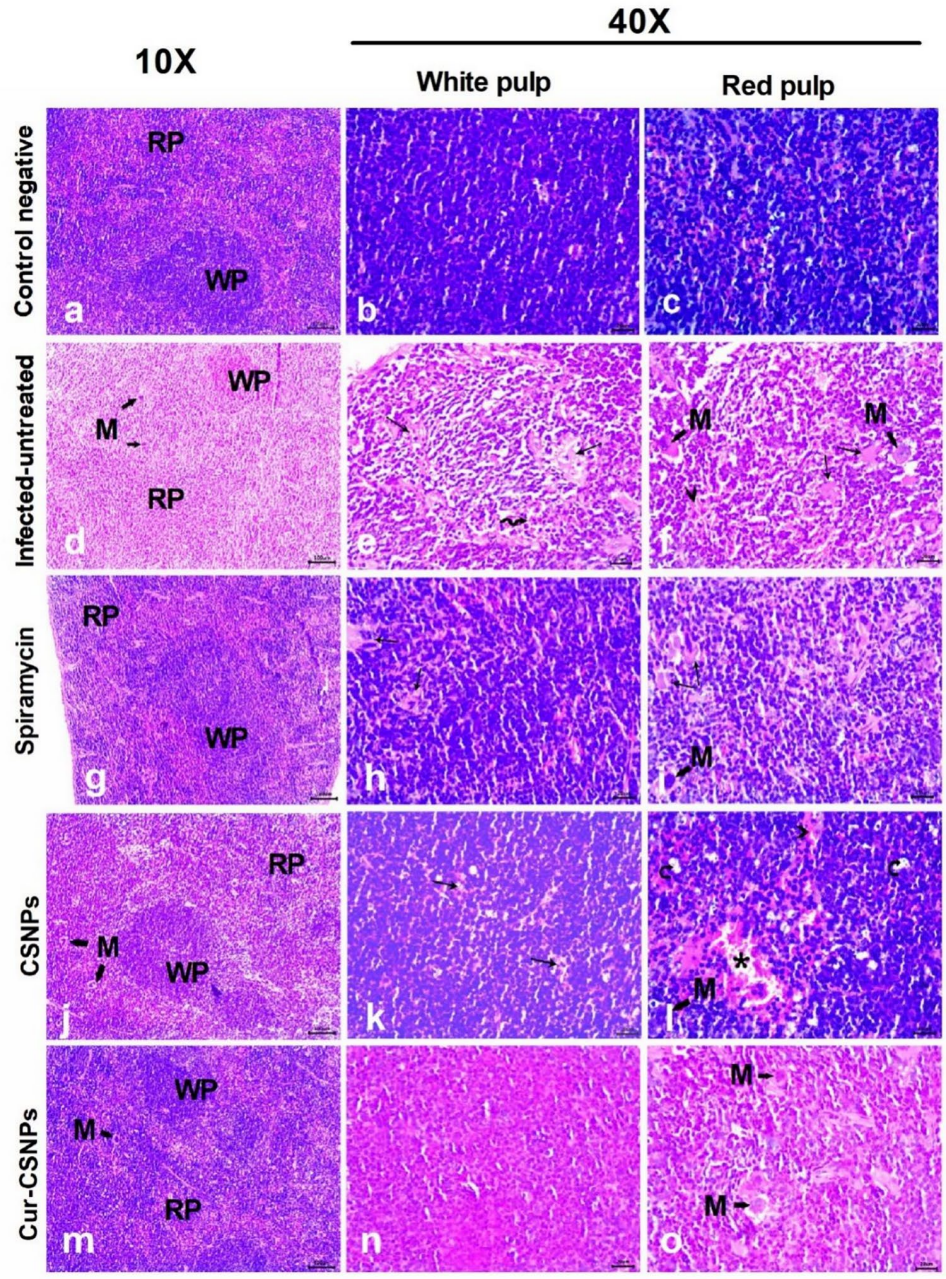
Photomicrograph of spleen tissue sections (n = 6 samples/group) stained with hematoxylin and eosin from negative control (**A**–**C**), infected-untreated (**D**–**F**), Spiramycin-treated (**G**–**I**), CSNPs-treated (**J**–**L**), and Cur-CSNPs-treated groups (**M**–**O**). (RP) red pulp, (WP) white pulp, (M) megakaryocytes, (arrow) lymphoid necrosis, (curved arrow) lymphoid depletion, (arrowhead) congestion, (wavy arrow) perifollicular hemorrhage, and (asterisk) hemorrhage in red pulp. CSNPs: Chitosan nanoparticles-treated group; Cur-CSNPs: curcumin-loaded Chitosan nanoparticles-treated group.

Table 3 presents the histopathological lesions scores for brain, liver, and spleen in the different groups. The brain in the negative control group had a normal architecture with intact neurons and glial cells (Fig. 6 a and b). In the infected untreated mice, the brain showed severe vascular congestion along with perivascular inflammatory cell infiltration, focal mononuclear cell infiltration, widespread neuronal degeneration  (Fig. 6c), gliosis (Fig. 6d), and multiple tissue cysts of *T. gondii* with bradyzoites (Fig. 6e). Furthermore, meningitis and severe meningeal hemorrhage were observed (Fig. 6f). The Spiramycin®-treated group showed preserved brain structure with mild vascular congestion and neuronal necrosis in some examined sections (Fig. 7 a and b). The CSNPs-treated group displayed moderate congestion and neuronal necrosis as well as parasitic tissue cysts in some examined sections (Fig. 7c and d), however, the Cur-CSNPs-treated group showed normal brain histoarchitecture (Fig. 7 e and f). The liver in the negative control group demonstrated a normal appearance of the hepatic architecture, blood vessels, and portal area (Fig. 8 a and b). In the infected untreated group, the liver showed remarkable changes in almost entire lobules, severe congestion, and perivascular infiltration of mononuclear cells (Fig. 8 c and d). Moreover, diffuse hepatocyte vacuolar degeneration and multifocal areas of coagulative necrosis infiltrated with mononuclear cells were noticed. Furthermore, there were multiple *T. gondii* tissue cysts and marked Kupffer cell reaction (Fig. 8 e and f). The Spiramycin®-treated group revealed normal hepatic parenchyma with preserved hepatocytes arrangement and portal triad structures (Fig. 9 a and b). Some lobules showed slight infiltration of mononuclear cells in portal areas (Fig. 9 b). Few tissue cysts and vascular congestion with perivascular inflammatory cell infiltration were observed in the mice treated with CSNPs (Fig. 9 c and d), whereas normal architecture of the hepatic parenchyma with mild vascular congestion was observed in the Cur-CSNPs (Fig. 9e and f). The spleen of the negative control group exhibited normal histomorphology of white and red pulps with typical cellular population and stromal tissues (Fig. 10a–c). The infected untreated group showed disruption in the splenic architecture with reduced cellularity with lymphoid necrosis in both white and red pulps (Fig. 10d–f). Perifollicular hemorrhage, congestion in the red pulp, and intense infiltration of megakaryocytes were also observed (Fig. 10d–f). The Spiramycin®-treated group exhibited normal white and red pulps structures with mild infiltration of megakaryocytes and lymphoid necrosis (Fig. 10g–i). The CSNPs-treated group exhibited mild necrosis and depletion of the lymphoid elements in white and red pulps as well as congestion and hemorrhagic necrotic foci in red pulp (Fig. 10j-l). However, the Cur-CSNPs-treated group had a normal white and red pulps appearance with mild megakaryocytes infiltration (Fig. 10 m–o).

**Table 3:** Score of severity and incidence of histopathological lesions in brain, liver, and spleen of all groups (n = 6 specimens/group).

Lesion	**Severity **^**a**^** (Incidence **^**b**^**) of lesions in all groups**				
	Negative control	Infected − untreated	Spiramycin	CSNPs	Cur − CSNPs
*Brain:* Congestion	− (0%)	+ + + (100%)	+ (83.3%)	+ + (83.3%)	− (0%)
Mononuclear cells infiltration	− (0%)	+ + + (100%)	− (0%)	+ (50%)	− (0%)
Neuronal degeneration	− (0%)	+ + + (100%)	+ (50%)	+ + (50%)	− (0%)
Gliosis	− (0%)	+ + (100%)	− (0%)	− (0%)	− (0%)
Meningitis	− (0%)	+ + + (83.3%)	− (0%)	− (0%)	− (0%)
Tissue cysts of *T. gondii*	− (0%)	+ + + (100%)	− (0%)	+ (50%)	− (0%)
*Liver:* Vascular congestion	− (0%)	+ + + (100%)	− (0%)	+ + (66.7%)	+ (50%)
Inflammatory infiltration	− (0%)	+ + + (100%)	+ (66.7%)	+ + + (83.3%)	− (0%)
Hepatocytes degeneration and necrotic foci	− (0%)	+ + + (100%)	− (0%)	− (0%)	− (0%)
Kupffer cells reaction	− (0%)	+ + (100%)	− (0%)	− (100%)	− (0%)
Tissue cysts of *T. gondii*	− (0%)	+ + (100%)	− (0%)	+ + (50%)	− (0%)
*Spleen:* Lymphoid necrosis and depletion in white pulp	− (0%)	+ + + (100%)	+ (83.3%)	+ (83.3%)	− (0%)
Lymphoid necrosis and depletion in red pulp	− (0%)	+ + + (100%)	+ (100%)	+ (83.3%)	− (0%)
Congestion in red pulp	− (0%)	+ + (83.3%)	− (0%)	+ (100%)	− (0%)
Hemorrhage	− (0%)	+ + (83.3%)	− (0%)	+ + (83.3%)	− (0%)
Infiltration of megakaryocytes	− (0%)	+ + + (100%)	+ (83.3%)	+ (100%)	+ (90%)

^a^ Score of lesion severity: ( −) absence of the lesion = 0%, ( +) mild = 5–25%, (+ +) moderate = 26–50%, and (+ + +) severe ≥ 50% of the examined tissue sections.

^b^ Percentage of mice showed lesion from total inspected. CSNPs (chitosan nanoparticles), Cur-CSNPs (curcumin-loaded chitosan nanoparticles).

### Immunohistochemical analysis of IL-6 and TNF-α in brain, liver, and spleen

**Fig. 11:**
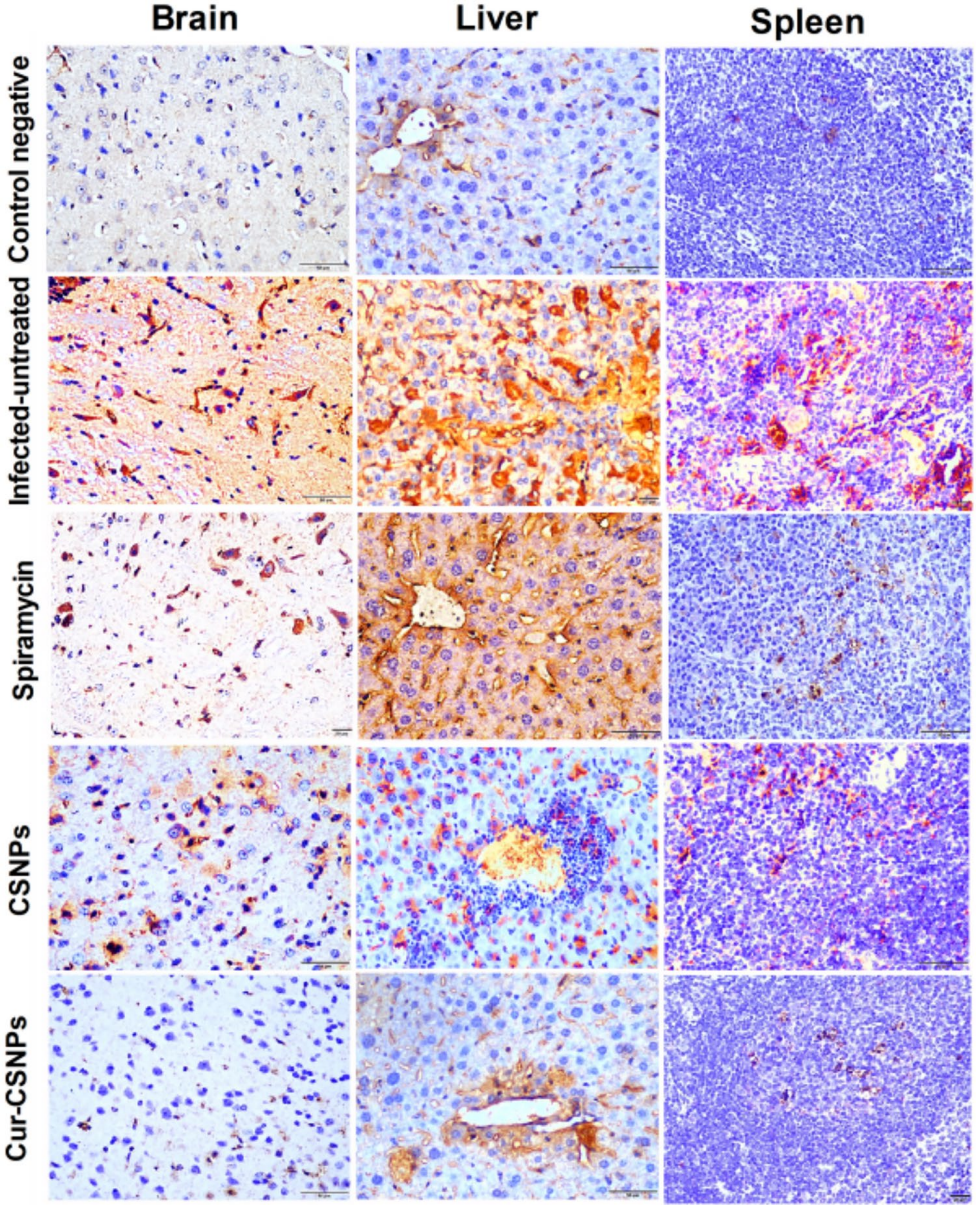
Immuno-expression of IL-6 in brain, liver, and spleen tissues. The anti-IL-6 antibodies demonstrate a strong and extensive, brown-colored expression in brain, liver, and spleen of the infected-untreated mice compared to their moderate expression in Spiramycin- and CSNPs- treated groups and weak expression in negative control and Cu-CSNPs-treated group. CSNPs: Chitosan nanoparticles-treated group; Cur-CSNPs: curcumin-loaded Chitosan nanoparticles-treated group.

**Fig. 12:**
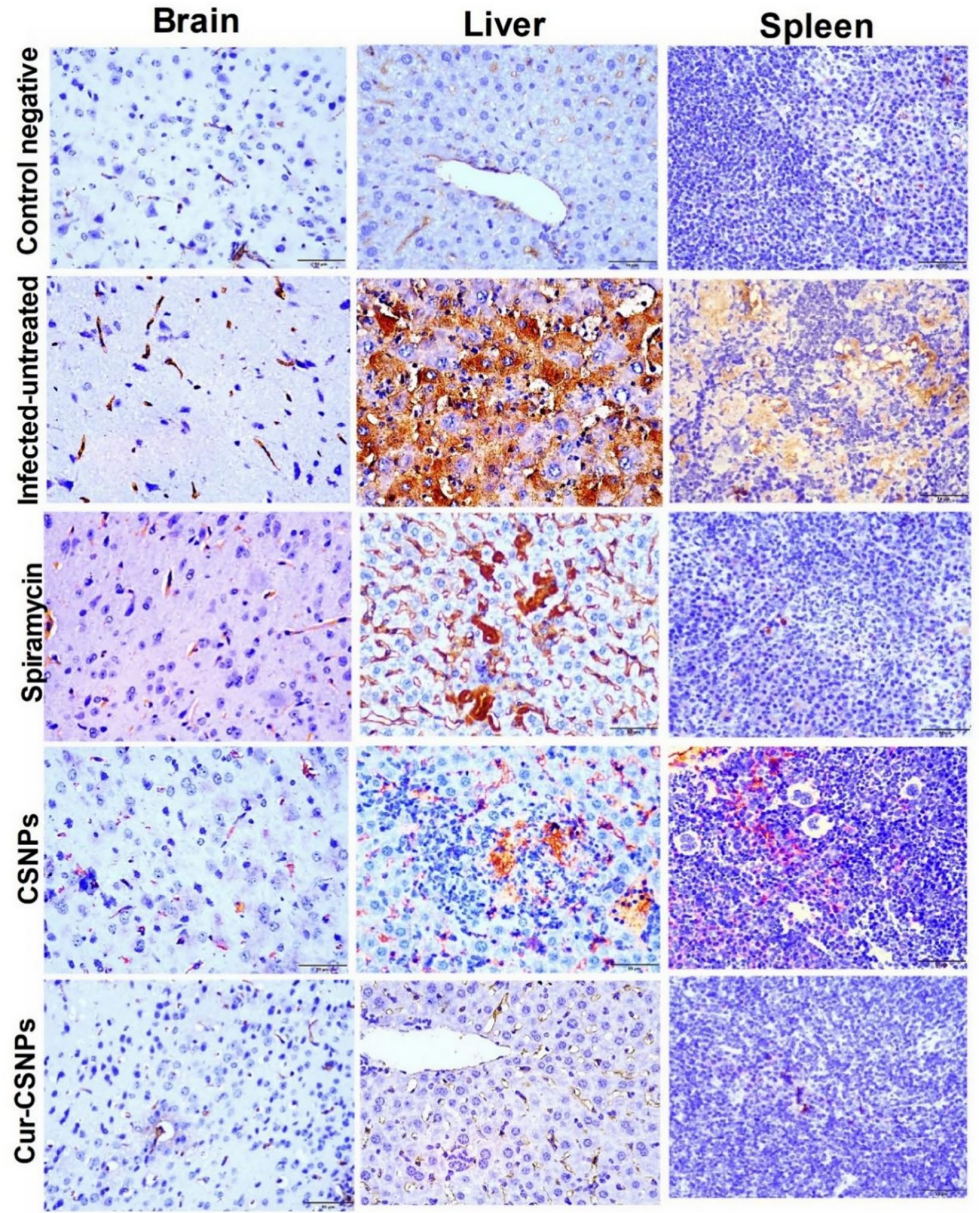
Immuno-expression of TNF-α in brain, liver, and spleen tissues. The anti-TNF-α antibodies demonstrate a strong and extensive, brown-colored expression in brain, liver, and spleen of the infected-untreated mice compared to their moderate expression in Spiramycin- and CSNPs- treated groups and weak expression in control negative and Cu-CSNPs-treated group. CSNPs: Chitosan nanoparticles-treated group; Cur-CSNPs: curcumin-loaded Chitosan nanoparticles-treated group.

Table 4 shows the percentage area of immunoreaction for anti-IL-6 and TNF-α antibodies in brain, liver, and spleen sections in the different groups. Brain, liver, and spleen tissue in the infected untreated group showed statistically significant upregulation in the expression of IL-6 and TNF-α compared to the negative control mice. A statistically significant decrease in the immunoreaction percentage area of IL-6 and TNF-α in the brain, liver, and spleen tissues was observed in all treated groups compared to the infected untreated group (Figs. 11 and 12).

**Table 4:** Area percentage of immunostaining reaction of IL-6 and TNF-α in the brain, liver, and spleen of the different groups.

Groups	IL-6			TNF-α		
	Brain	Liver	Spleen	Brain	Liver	Spleen
Control negative	4.04 ^b^ ± 0.49	11.8 ^c^ ± 1.2	2.5 ^b^ ± 0.7	0.41^b^ ± 0.1	2.6 ^d^ ± 0.25	1.96 ^b^ ± 0.25
Infected-untreated	15.1 ^a^ ± 2.79	25 ^a^ ± 0.4	12.3 ^a^ ± 1.9	4.1 ^a^ ± 1.1	30.5 ^a^ ± 2.6	10.6 ^a^ ± 1.8
Spiramycin	7.8 ^b^ ± 0.39	19.6 ^b^ ± 1.6	6.4 ^b^ ± 1.9	1 ^b^ ± 0.3	24.2 ^b^ ± 1.92	5.99 ^b^ ± 1.1
CSNPs	6.9 ^b^ ± 1.1	10.3 ^c^ ± 0.9	5.8 ^b^ ± 0.7	0.8 ^b^ ± 0.4	9.6 ^c^ ± 0.6	3.5 ^b^ ± 1.5
Cur-CSNPs	4.9 ^b^ ± 1.41	9.5 ^c^ ± 0.5	3.9 ^b^ ± 1.2	0.8 ^b^ ± 0.1	5.8 ^dc^ ± 1.1	2.8 ^b^ ± 1.1
*P* value	0.003	0.001	0.005	0.004	0.001	0.004

^a,b,c,d^indicates significant difference between groups (*P* < 0.05). CSNPs (chitosan nanoparticles), Cur-CSNPs (curcumin-loaded on chitosan nanoparticles). IL-6: Interleukin-6, TNF-α: Tumor Necrosis Factor Alpha.

## Discussion

This study evaluated the therapeutic potential of chitosan nanoparticles (CSNPs) and curcumin-loaded chitosan nanoparticles (Cur-CSNPs) in treating chronic *Toxoplasma gondii* infection, with particular attention to their physicochemical properties, antioxidant activity, and immunomodulatory effects. A critical determinant of nanoparticle performance is particle size and surface charge (zeta potential), influencing stability, bioavailability, and tissue penetration.

CSNPs typically exhibited strong positive zeta potential in the range of + 40 to + 50 mV, attributed to the protonated amino groups on the cationic surface of chitosan, ensuring its colloidal stability, reducing the risk of particle aggregation, supporting prolonged systemic circulation^46^, and suitability for biomedical applications through electrostatic repulsion^47^. Moreover, CSNPs exhibited nanoscale dimensions (~ 22.68 nm and 1.39 nm), facilitating enhanced cellular uptake via endocytosis and deeper tissue penetration. However, Cur-CSNPs exhibited larger particle sizes (~ 623.1 nm and 169.6 nm), likely due to the encapsulation of curcumin, a hydrophobic compound with low aqueous solubility, which is consistent with previous studies reporting size enlargement following incorporation of hydrophobic drugs^48^. Despite their larger size, the nanoformulation successfully improved curcumin’s bioavailability and therapeutic profile, consistent with earlier findings^10,49^. The zeta potential of Cur-CSNPs decreased to + 19.0 mV, indicating successful curcumin encapsulation and neutralizing the positive surface charge reported for chitosan-based carriers^47^. These cationic surface charges promote electrostatic interactions with negatively charged cell membranes and microbial surfaces, facilitating adhesion and internalization^50^. Moreover, the reduction of zeta potential of Cur-CSNPs maintains colloidal stability (> ± 15 mV) while minimizing the risk of hemolytic effects from high ZP (> + 30 mV), which could disrupt negatively charged erythrocyte membranes^51^. In our study, no significant hemolysis was observed for CSNPs or Cur-CSNPs at used therapeutic concentrations, supporting their biocompatibility for *T. gondii* treatment. Thus, curcumin loading not only enhances the therapeutic efficacy of CSNPs but also improves their safety profile^52^. The PDI of Cur-CSNPs increased to 0.93 compared to CSNPs, reflecting greater heterogeneity in size distribution, this outcome is typical when embedding hydrophobic molecules into hydrophilic matrices. The increased particle size can be attributed to the structural complexity of curcumin and its high encapsulation efficiency (95.6%), which also reflects a stable and effective formulation^53^. These properties are particularly advantageous for crossing physiological barriers, such as the blood–brain barrier (BBB), a crucial consideration in management cerebral toxoplasmosis^54^. The efficacy of Cur-CSNPs in crossing the BBB can be attributed to several complementary mechanisms. First, the moderately positive surface charge (+ 19 mV) promotes adsorptive-mediated transcytosis via electrostatic interactions with negatively charged endothelial membranes. Additionally, chitosan can transiently modulate tight junction proteins, enhancing paracellular transport without causing permanent BBB disruption^52^. The nanoscale size (307 nm) and amphiphilic properties of Cur-CSNPs further facilitate cellular uptake and transcytosis, improving brain delivery^55^. These features together enable efficient transport and neuroprotective potential of curcumin against *T. gondii* in the central nervous system (CNS)^56^. These findings are consistent with recent studies demonstrating that nanoparticle surface charge, size, and tight junction modulation are key factors in overcoming BBB limitations for effective CNS drug delivery.

Molecular analysis confirmed that the strain used in this study belongs to the *T. gondii* Type II clonal lineage, one of the most common and widely distributed lineages. This lineage is typically associated with chronic infection and moderate virulence. Although *T. gondii* strains share core genetic features, they elicit different host immune responses, which may influence disease outcome and the efficacy of therapeutic interventions^57^ [32,33]. Phylogenetic analysis further supported the classification of the strain within the Type II group and highlighted its genetic similarity to other regional isolates^58^.

In the experimental infection, both the CSNPs- and Cur-CSNPs-treated groups demonstrated promising therapeutic outcomes, evidenced by a reduction in both the size and number of *T. gondii* brain cysts in infected animals. These findings align with prior studies demonstrating the efficacy of curcumin and chitosan nanoparticles in reducing parasitic burden^59,60^. Particularly noteworthy is the Cur-CSNPs-treated group, which exhibited the highest percentage reduction, indicating that encapsulating curcumin within chitosan nanoparticles enhances its ability to inhibit the growth and maturation of *Toxoplasma* cysts^61^.

The cyst wall is a critical structure protecting *Toxoplasma* from host immune responses and environmental stress, contributes to the parasite’s persistence and chronicity^62^. Our findings revealed that cyst walls in the infected untreated and Spiramycin®-treated groups appeared more regular and intact, indicative of normal maturation and development^63^. In contrast, the CSNPs and Cur-CSNPs-treated groups showed cysts with disrupted or damaged walls, suggesting that these treatments may compromise cyst integrity. These findings are consistent with previous studies showing that natural product-based treatments can alter *T. gondii* cyst morphology and potentially reduce its persistence^64^.

Hematological analyses provided insights into the systemic repercussions of the treatments. Our results reveal a significant reduction in total WBC counts in the CSNPs and Cur-CSNPs-treated groups compared to the infected untreated group and Spiramycin-treated group, indicating general anti-inflammatory effects. However, the CSNPs and Cur-CSNPs-treated groups showed a statistically significant decrease in the percentage of lymphocyte and non-significant decreases in the percentage of neutrophils, monocytes, and eosinophils. These results concurred with previous literatures^10,35,65^, highlighting the nuanced immunomodulatory profiles of each treatment, influencing immune cell populations variably, and suggesting potential tailored therapeutic applications. Moreover, the reduction in total leukocyte counts associated to the mechanism of transcellular diffusion of protozoans to CNS^33^.

Interestingly, our results demonstrated that the Cur-CSNPs and CSNPs-treated groups exhibited no significant differences in total antioxidant capacity (TAC) compared to the negative control group. In contrast, the infected untreated group and Spiramycin®-treated group exhibited a significant decrease in TAC, emphasizing the importance of therapeutic interventions in restoring antioxidant defense during infection. On the other hand, malondialdehyde levels (MDA) were significantly increased in the infected untreated group, indicating increased lipid peroxidation and oxidative stress^66^. However, no significant differences were observed in the Cur-CSNPs and CSNPs-treated groups, suggesting their effectiveness in mitigating lipid peroxidation, and maintaining oxidative balance. These findings underscore the potential of Cur-CSNPs and CSNPs as therapeutic agents in alleviating oxidative stress and preserving antioxidant status during *T. gondii* infection, consistent with previous in vivo and clinical studies^67^.

Histopathological examination of brain, liver, and spleen tissues provided valuable insights into the impact of *T. gondii* infection and treatment efficacy. Our results revealed that the infected untreated mice exhibited severe *T. gondii*-related changes, including vascular congestion, inflammatory cell infiltration, neuronal degeneration, gliosis, meningitis, and tissue cysts. These findings align with the neurotropic nature of *T. gondii* and its potential to cause neurological manifestations^68^. Toxoplasmic encephalitis, a serious complication of *T. gondii* infection, characterizes by the parasite’s ability to invade and persist within the CNS. Upon entry into the body, *T. gondii* can breach the blood–brain barrier, establishing itself in the brain and spinal cord, which may remain dormant or reactivate, leading to further neurological damage^69^. The Spiramycin®-treated group partially improved lesions, with residual congestion and necrosis, suggesting limited therapeutic effect in mitigating brain tissue damage^70^. On the other hand, treatment with CSNPs showed moderate improvement, whereas Cur-CSNPs remarkably preserved brain histoarchitecture, suggesting potential synergy between curcumin and CSNPs in mitigating *T. gondii*-induced damage^71,72^. Curcumin has anti-inflammatory and neuroprotective properties, could enhance the therapeutic efficacy of CSNPs in combating *T. gondii* infection in the brain^59^. It acts by suppressing inflammatory pathways, reducing oxidative stress, and promoting neuronal survival and regeneration. Specifically, curcumin modulates key molecular targets involved in neuroprotection, such as NF-κB, MAPKs, and various antioxidant enzymes, thereby protecting brain cells from damage and supporting their function in neuroinflammatory conditions^73^.

Spleen and liver unveiled significant findings in the different groups compared to the negative control group. The liver of the infected untreated group showed severe histopathological alterations, encompassing vascular congestion, inflammatory infiltration, and the presence of bradyzoites amidst hepatic tissue, alongside hepatocyte degeneration and necrotic foci, as previously reported by Barakat, et al.^74^. These observations underscore the profound impact of the infection on hepatic function and integrity. Noteworthy, treatment with Spiramycin® and CSNPs-treated groups showed restored hepatic architecture, albeit with residual inflammatory changes noted in the CSNPs-treated group^75^. Intriguingly, Cur-CSNPs-treated group exhibited minimal histologic abnormalities^71^, implying a potential therapeutic edge over alternative treatments.

In spleen, the negative control group portrayed a normative histomorphology, characterized by well-maintained white and red pulp structures, featuring a balanced cellular composition and intact stromal tissues^76^. The infected untreated group exhibited marked alterations in splenic architecture, lymphoid necrosis, depletion, congestion, and hemorrhage, underscoring the systemic repercussions of the infection on immune function^77^. The Spiramycin® and CSNPs-treated groups partially alleviated these changes, with mild histologic abnormalities in the CSNPs-treated group, indicating limitations in fully restoring splenic morphology^35^. The Cur-CSNPs-treated group exhibited conserved appearance of white and red pulps with mild infiltration of megakaryocytes, suggesting potential therapeutic advantages. These findings align with previous findings from an in vivo antileishmanial study, which demonstrated significantly enhanced suppression of parasite replication in the spleen with curcumin-loaded nanoparticles.^78^. In the present study, the CSNPs and Cur-CSNPs significantly downregulated IL-6 and TNF-α expression in brain, liver, and spleen, indicating potential immunomodulatory effects and anti-inflammatory properties.

The Spiramycin®-treated group showed elevated IL-6 expression and limited efficacy in modulating TNF-α expression, suggesting potential exacerbation of inflammation^79,80^. These results underscore the importance for alternative therapeutic agents that effectively modulate inflammatory pathways. Chitosan and curcumin nanoparticles hold promise as novel therapeutic agents^48^, with previous findings supporting their protective effects in attenuating inflammation and enhancing angiogenesis^81^. The therapeutic effects of Cur-CSNPs, including reduced parasite burden and attenuated inflammatory responses, were comparable to those of Spiramycin®. However, this similarity in efficacy should not obscure the potential advantages of Cur-CSNPs as a natural, biocompatible, and cost-effective alternative. Curcumin, derived from a dietary source, is known for its favorable safety profile, limited side effects, and antioxidant and immunomodulatory properties. Its formulation within chitosan nanoparticles may enhance bioavailability and therapeutic impact, offering a promising approach for treating chronic toxoplasmosis while minimizing reliance on chemical medications that may carry higher toxicity or resistance risks. Thus, our study supports the potential integration of natural compounds like Cur-CSNPs into antiparasitic treatment strategies, particularly in resource-limited or ecologically sensitive contexts.

The CSNPs and Cur-CSNPs exhibited strong therapeutic efficacy against *T. gondii* by modulating oxidative stress and regulating immune responses. The infection induced marked oxidative imbalance, evidenced by elevated malondialdehyde level, and reduced total antioxidant capacity. Treatment with CSNPs, and more prominently, Cur-CSNPs, significantly restored TAC and reduced MDA concentrations, indicating a reinforced antioxidant defence system^82,83^. These improvements can be attributed to the synergistic antioxidant effects of curcumin and chitosan. Curcumin is known to scavenge reactive oxygen species (ROS), inhibit nitric oxide (NO) production, and activate the Nrf2 signalling pathway^84^ , while chitosan enhances the activity of endogenous antioxidant enzymes^18^. Concurrently, both nanoformulations effectively suppressed pro-inflammatory cytokines, particularly IL-6 and TNF-α, by inhibiting NF-κB, a key mediator of inflammation^85^. Notably, the Cur-CSNPs demonstrated superior immunomodulatory effects by downregulating Th17 cell responses and promoting regulatory T cell (Treg) activity, contributing to immune homeostasis^86^. Previous studies also support the role of curcumin and chitosan in reducing the expression of IL-1β, IL-6, IL-8, and TNF-α, reinforcing their anti-inflammatory synergy^48,80^. Nanoencapsulation enhances the bioavailability, stability, and targeted delivery of these compounds, offering a comprehensive therapeutic strategy to reduce parasite burden and mitigate tissue damage associated with chronic *Toxoplasma* encephalitis^10,81^.

## Conclusion

Cur-CSNPs and CSNPs showed significant therapeutic efficiency against chronic *T. gondii* infection. The Cur-CSNPs-treated animals revealed a notable reduction in *T. gondii* brain cyst size and number with maintain brain, liver, and spleen architecture, as well antioxidant balance. These findings highlight Cur-CSNPs as a promising, biocompatible alternative to Spiramycin for managing chronic toxoplasmosis. Although morphological alterations in the cyst wall were observed, parasite replication or viability were not directly assessed; therefore, interpretations regarding the inhibitory effects on parasite development remain speculative. Nonetheless, we acknowledge that distinguishing between these two effects requires further investigation, such as evaluating cytokine levels at earlier time points or confirm intercellular biodistribution of nanoparticles.

## Supplementary Information

Below is the link to the electronic supplementary material.

Supplementary Information 1.

## Data Availability

The dataset(s) supporting the conclusions of this article is included within the article and its additional file(s).
