# Supplementary material for: Exploring the role of chitosan and curcumin-loaded chitosan nanoparticles against chronic toxoplasma infection in experimental mice
Source: Sci Rep. 2025 Nov 24;15:41765. doi: 10.1038/s41598-025-25252-5 (PMC12647587; doi:10.1038/s41598-025-25252-5)
Supplement: Supplementary file 1 — Supplementary Information 1. [file 41598_2025_25252_MOESM1_ESM.docx]

**Pilot Study**

In this preliminary experiment, three groups of infected mice (n = 3 per group) were treated with different doses of Cur-CSNPs: 0.5 mL, 1 mL, and 1.5 mL per mouse for 10 days to observe immediate clinical signs, survival, or efficacy markers. We observed mortality in the group receiving the highest dose (1.5 mL), while the 0.5 mL group showed the best safety profile and acceptable therapeutic response.

**Table S1:** Pilot study assessing the safety and efficacy of different Cur-CSNPs doses in experimentally infected mice:

| **Group** | **Dose of Cur-CSNPs** | **No. of Mice** | **Mortality** | **Observation** |
| --- | --- | --- | --- | --- |
| A | 0.5 mL | 3 | 0/3 | Well-tolerated, mild efficacy |
| B | 1.0 mL | 3 | 1/3 | Slight distress, moderate efficacy |
| C | 1.5 mL | 3 | 2/3 | Toxic effects, high mortality |


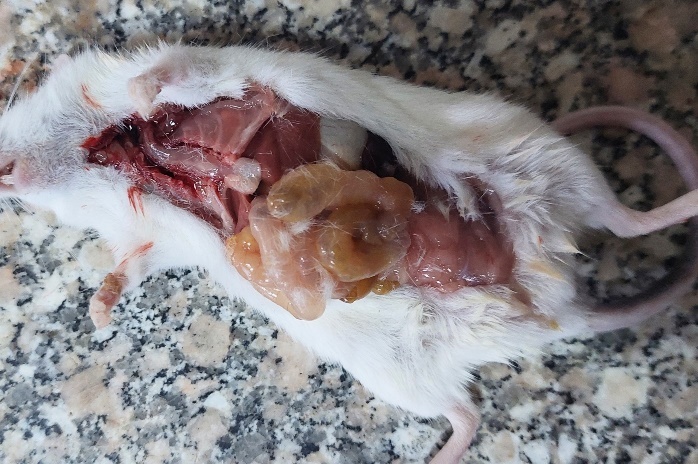


**Figure S1**: Death of mice treated with 1 mL of Cur-CSNPs observed 4 days post-administration, showing intestinal bloating upon evisceration.

Based on these findings, we selected 0.5 mL as the optimal and safe dose for use in the main experiment. These details, including the observed mortality.


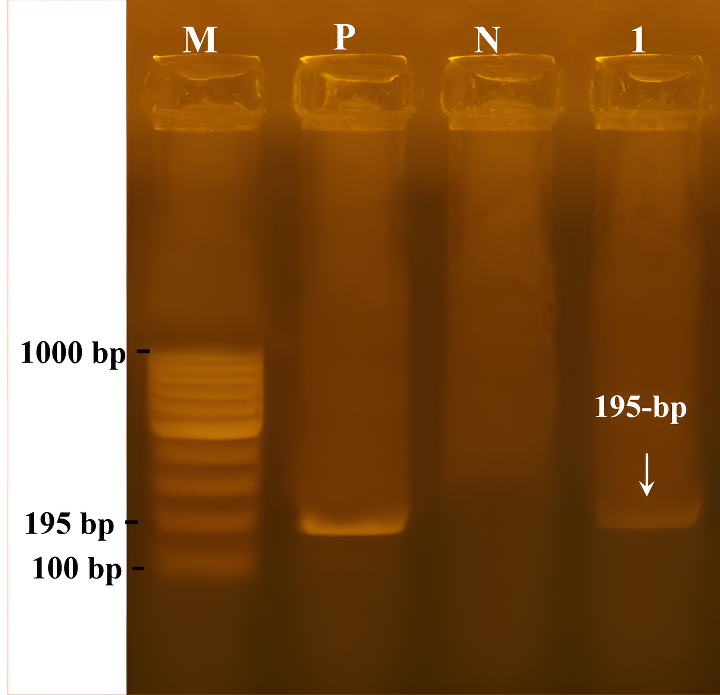


**Figure S2:** Agarose (1.5%) gel showing the PCR products (195-bp) of amplified from positive samples. Lane (M) is DNA size marker, lane (P) is positive control, lane (N) is negative control, Lane (1) is the positive sample.


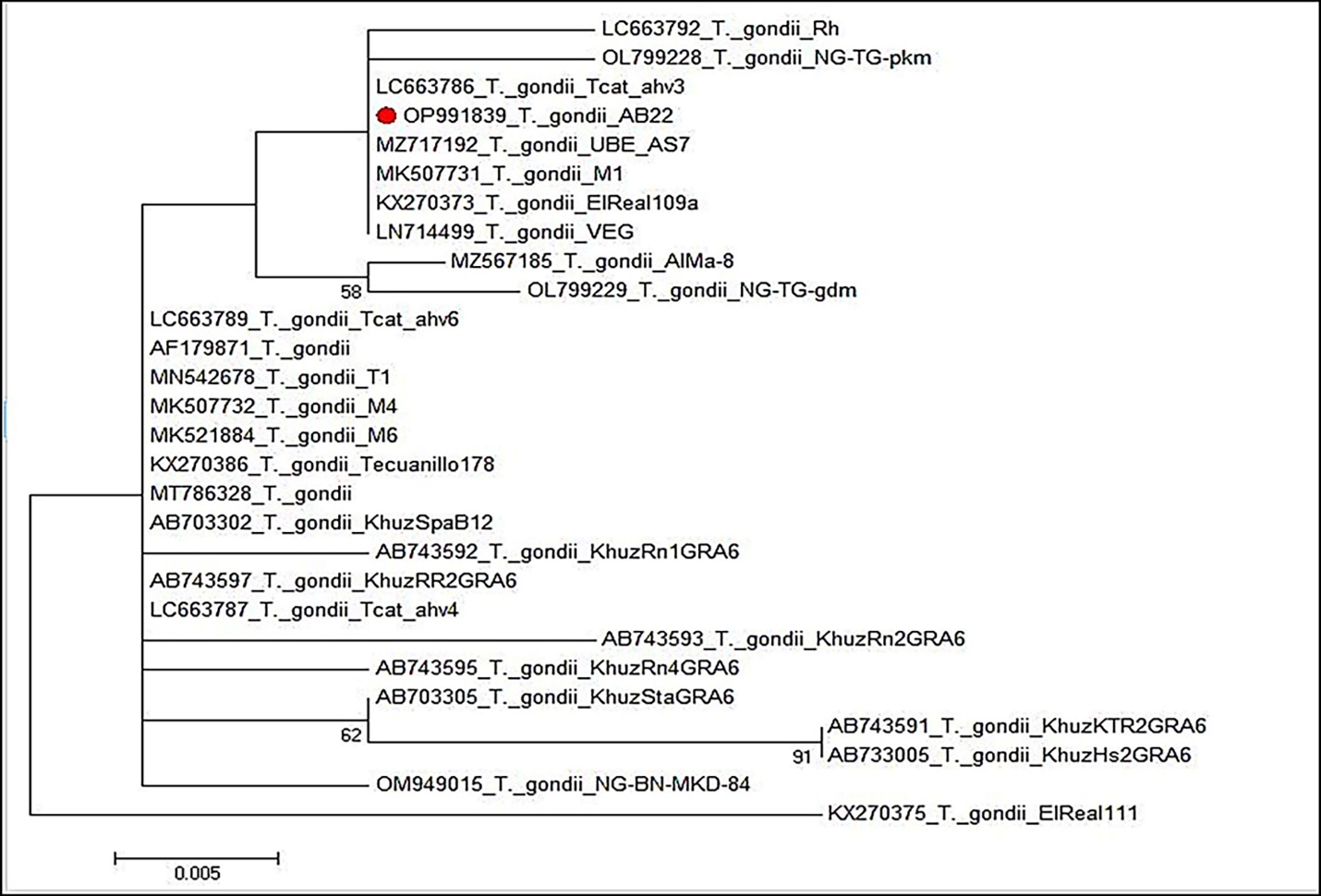


**Figure S3:** Portrays a phylogenetic tree constructed from B1 gene sequences, revealing distinct clades that signify discernible genetic lineages within Toxoplasma gondii. The bar denoting evolutionary distance divergence represents 0.005 substitutions per site.
